# The closed-edge structure of graphite and the effect of electrostatic charging†

**DOI:** 10.1039/c9ra09913a

**Published:** 2020-02-24

**Authors:** Victor Posligua, Joana Bustamante, Cesar H. Zambrano, Peter J. F. Harris, Ricardo Grau-Crespo

**Affiliations:** Department of Chemistry, University of Reading Whiteknights Reading RG6 6AD UK r.grau-crespo@reading.ac.uk; Departamento de Química, Universidad Técnica Particular de Loja San Cayetano Alto Loja 1101608 Ecuador; Instituto de Simulación Computacional (ISC-USFQ), Universidad San Francisco de Quito Diego de Robles y Vía Interoceánica 17-1200-841 Quito Ecuador; Electron Microscopy Laboratory, University of Reading J. J. Thomson Building, Whiteknights Reading RG6 6AF UK

## Abstract

The properties of graphite, and of few-layer graphene, can be strongly influenced by the edge structure of the graphene planes, but there is still much that we do not understand about the geometry and stability of these edges. We present an experimental and theoretical study of the closed edges of graphite crystals, and of the effect of an electric field on their structure. High-resolution transmission electron microscopy is used to image the edge structure of fresh graphite and of graphite that has been exposed to an electric field, which experiences a separation of the graphene layers. Computer simulations based on density functional theory are used to rationalise and quantify the preference for the formation of multiple concentric loops at the edges. A model is also presented to explain how the application of an electric field leads to the separation of the folded edges.

## Abstract 2

DFT simulations unravel the thermodynamics of folded edges in graphite, and explain why an electric field can open the loops.

## Graphical abstract

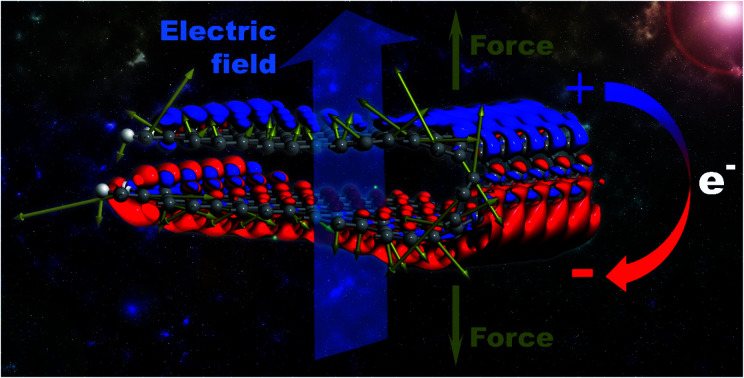

## 1. Introduction

Much of what we know about the microstructure of carbon materials has come from studies using transmission electron microscopy (TEM).^1^ Often, such studies have shown that the simple textbook pictures that we have of graphite, diamond and other forms of carbon need to be modified. For example, graphite is traditionally portrayed as consisting of individual, unconnected graphene layers stacked one above the other, but TEM has shown that in reality the graphene sheets are frequently joined at the edges by arched structures which encompass two or more adjacent layers, forming “closed” edges.^2–4^

The formation of closed edges in graphitic structures has important consequences, which makes them the subject of research interest. First, the edge structure can influence the optical, magnetic, electrical, and electronic properties of graphite and of few-layer graphene.^5,6^ A theoretical study by Yan *et al.*^7^ has recently shown that AB-stacked closed-edge bilayer graphene exhibits band gap opening and charge separation, which suggests potential applications in electronic devices, such as solar cells. In some cases, closed edges in graphite may be undesirable, *e.g.* their presence can prevent the production of graphene by exfoliation of highly oriented pyrolytic graphite (HOPG). The experimental results presented in this paper were obtained using commercial graphite, produced by the high temperature treatment of coke, leading to a less perfect structure than that of HOPG. However, closed edges, as well as structural transformations due to electric charging, have also been reported for HOPG.^8^ Finally, another motivation to investigate the properties of closed edges is that they are structurally similar to collapsed nanotube structures,^9^ which have increasing technological relevance.^10^ It is known that multi-walled carbon nanostructures above a certain critical diameter (which depends on the number of walls^11^) tend to collapse and form nearly-flat structures with concentric loops at the edges. However, the analogy between graphitic closed edges and collapsed nanotubes is not exact, because in the former case the packing of the loops at the edges introduces a constraint in their diameters, which must match an integer number of interlayer distances, whereas in collapsed nanotubes “dog-bone” cross-sections are typically formed.

In this paper we first describe TEM studies of the edge structure of graphite, showing the formation of concentric loops. We then report images of graphite samples that have been exposed to an electric field. It is now well established that electric fields can transform the structure of carbon materials^8,12–21^ but the mechanism of the transformation is poorly understood. An earlier study suggested that the mechanism involved, in part, a separation of the closed graphite edges. We study the separation process in detail and use high-tilt imaging to determine the overall shapes of the transformed structures. We also present a theoretical analysis, based on density functional theory (DFT) simulations, of the thermodynamics of closed edges and the effect of an electric field, in order to interpret the observed TEM images. Previous theoretical work^22^ has focused on the geometry and electronic structure of closed-edge graphitic nanoribbons, without consideration of their relative stabilities. Here, we have calculated the variation of the surface energy of the edge as a function of the number of concentric folds, demonstrating that the formation of multiple folds is thermodynamically favoured. We also elucidate the mechanism by which an electric field expands the closed edges, explaining the structural transformations observed in experiment.

## 2. Methods

### 2.1. Experimental

The graphite used in the experiments consisted of commercial synthetic graphite rods obtained from Quorum Technologies Ltd, UK. Samples of the fresh rods were prepared for TEM by grinding in an agate mortar under isopropanol, mixing in an ultrasonic bath and depositing onto lacey carbon TEM grids. Edges of the graphite crystals were then imaged using a JEOL 2010 microscope, with a point resolution of 0.19 nm, operated at 200 kV. At this accelerating voltage there is a danger of irradiation damage,^23^ since the threshold for knock-on damage is well below 200 kV. Experiments carried out with the samples studied here showed that visible damage occurred only after about 2 min exposure to a beam with a current density of 15 pA cm^−2^. Care was taken not to expose the carbon to an electron beam for longer than this time. In the ESI (Fig. S5†) we illustrate the effect of a very intense electron beam on the samples.

In order to pass a current through the graphite, we used a commercial arc-evaporator, which is normally used for carbon-coating specimens for electron microscopy. In this unit, the electrodes are graphite rods, one of which is thinned to a diameter of approximately 1.4 mm at the point of contact. The chamber is evacuated by a turbomolecular pump to a pressure of approximately 3 × 10^−4^ mbar. Before carrying out the ‘‘evaporation’’, the rods are out-gassed by passing a current of about 30 A for 1 min. Some samples of graphite which had just been exposed to the out-gassing step were examined by TEM and no obvious structural changes were observed. For evaporation, a current of 75 A is passed for 3 s. Following evaporation, the thinned carbon rod was found to have slightly shortened, and a small deposit was formed in the area where the two rods made contact. This was collected and imaged in the TEM as described above.

### 2.2. Theory

The Vienna *Ab initio* Simulation Package (VASP)^24,25^ was used to carry out the quantum mechanical calculations within the Kohn–Sham implementation of the density functional theory (DFT). It is well established that the generalized gradient approximation (GGA), which is widely used in DFT simulations, is inadequate for the description of long-range dispersion interactions,^26^ which play an important role in graphitic structures. Although the local density approximation (LDA) of DFT does lead to interlayer binding and is sometimes used to investigate graphitic and other layered structures, including in previous work on closed-edged structures,^22^ this approximation is also unable to provide an adequate description of long-range dispersion interactions.^18^ We therefore employ here a functional with non-local correlation (optPBE-vdW)^27,28^ which has been successfully used in modelling graphite and other layered materials.^29–32^ In contrast with functionals with an empirical correction added to describe dispersion, like those proposed by Grimme *et al.*,^33,34^ non-local correlation functionals allow for the charge density to respond to the dispersion interactions, providing a more robust description of interlayer interactions. This functional also gives a good description of the bending stiffness of graphene layers, which is necessary for investigating the thermodynamics of closed edges. By calculating nanotubes of increasing radii and plotting their energies *vs.* the square of their radii, we obtain a bending modulus *D* = 1.57 eV, in agreement with the experimental value of 1.7 ± 0.2 eV.^35^

Valence wavefunctions are expanded in terms of planewaves with kinetic energies up to 520 eV. The interaction of the valence electrons with the ionic cores is modelled using the projector augmented wave (PAW) method,^36,37^ with C 1s levels frozen at the atomic reference state. Equilibrium structures were found by energy minimization until the forces on all atoms were less than 1 meV Å^−1^. A Γ-centred grid of *k*-points was used for integrations in the reciprocal space, with points separated by no more than ∼0.02 Å^−1^ in periodic directions.

The surface energy (*γ*), which characterizes the stability of a particular surface termination (for the edges in this case), was calculated as:1
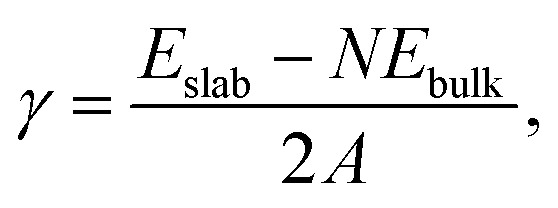
where *E*_slab_ is the energy per unit cell of a periodic slab with *N* carbon atoms and the graphitic edges pointing towards the vacuum gap, *E*_bulk_ is the energy per atom of bulk graphite, and *A* is the area per unit cell at each side of the slab. As VASP employs 3D periodic boundary conditions, all our models include vacuum regions of ∼10 Å separating the slabs, which were proved to be large enough not to affect the equilibrium geometries and surface energies.

In the simulations involving an electric field, we applied a periodic sawtooth-like potential perpendicular to the layers, as done by other authors (*e.g.*ref. 38). The induced charge redistribution is visualised as the difference in charge density with and without electric field.

## 3. Results and discussion

### 3.1. TEM of graphite edges

**Fig. 1:**
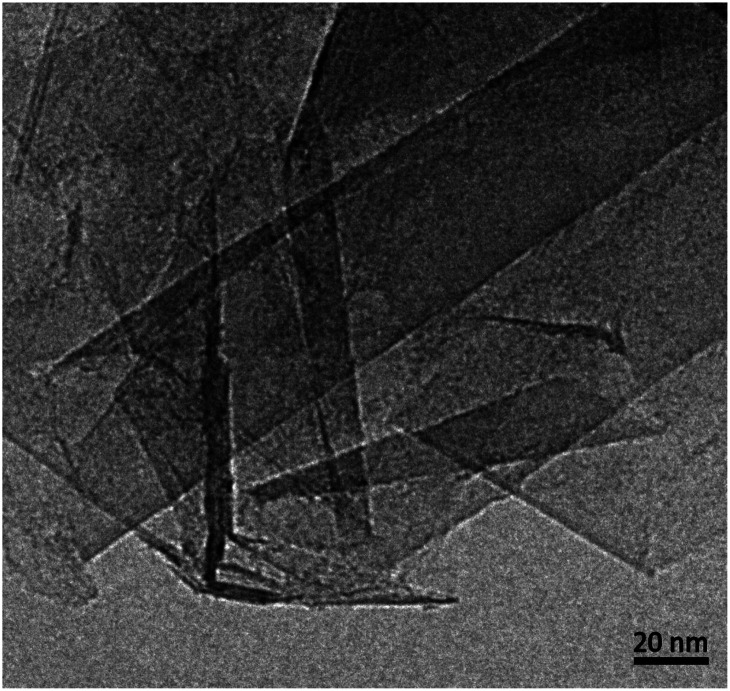
Transmission electron micrograph of carbon from fresh graphite rod.

A typical image of material from the fresh graphite is shown in Fig. 1. As expected, this consists mainly of flat crystallites, ranging from a few 100 nm to about 5 μm in size, containing up to 100 layers. The crystallites were often folded and buckled, and were covered with small amounts of finely-divided material. No nanotubes or other fullerene-related structures were seen in the fresh graphite. The graphite sheets tended to lie flat on the carbon support films, with the *c*-axis parallel to the electron beam. However, it was possible to find crystals orientated with the graphene planes parallel to the electron beam, and a number of images were recorded of the edges of such crystals. In some cases the edges were completely open, with no connections between adjacent layers, but in other cases the layers were clearly joined by arched structures.

**Fig. 2:**
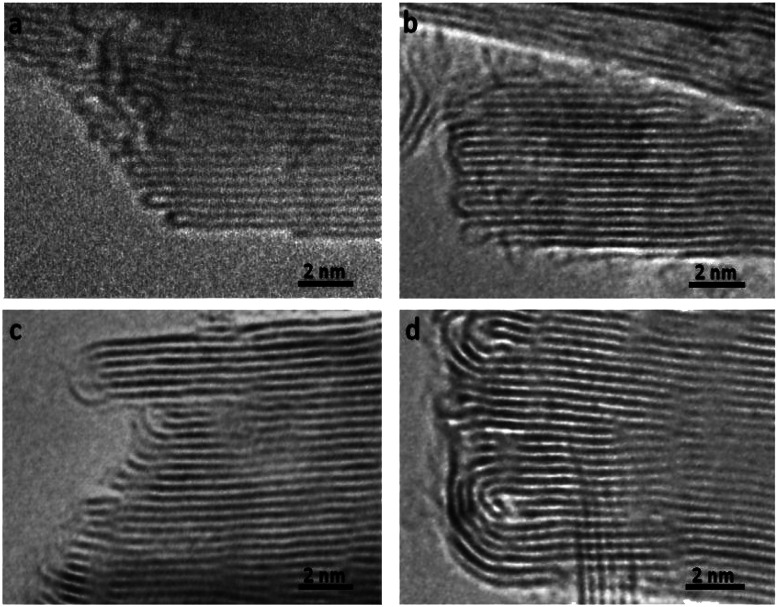
TEM images of looped edges in fresh graphite. (a) Edge with single loops, (b) edge with single and multiple loops (c) loops apparently in the process of being formed, (d) multiple loops.

Different edge structures were observed, as shown in Fig. 2. Single loops, as shown in Fig. 2a, were sometimes seen, but multiple loops, as in the other images, were more common. Fig. 2c appears to show some loops in the process of being formed.

### 3.2. Thermodynamics of closed edges from DFT calculations

**Fig. 3:**
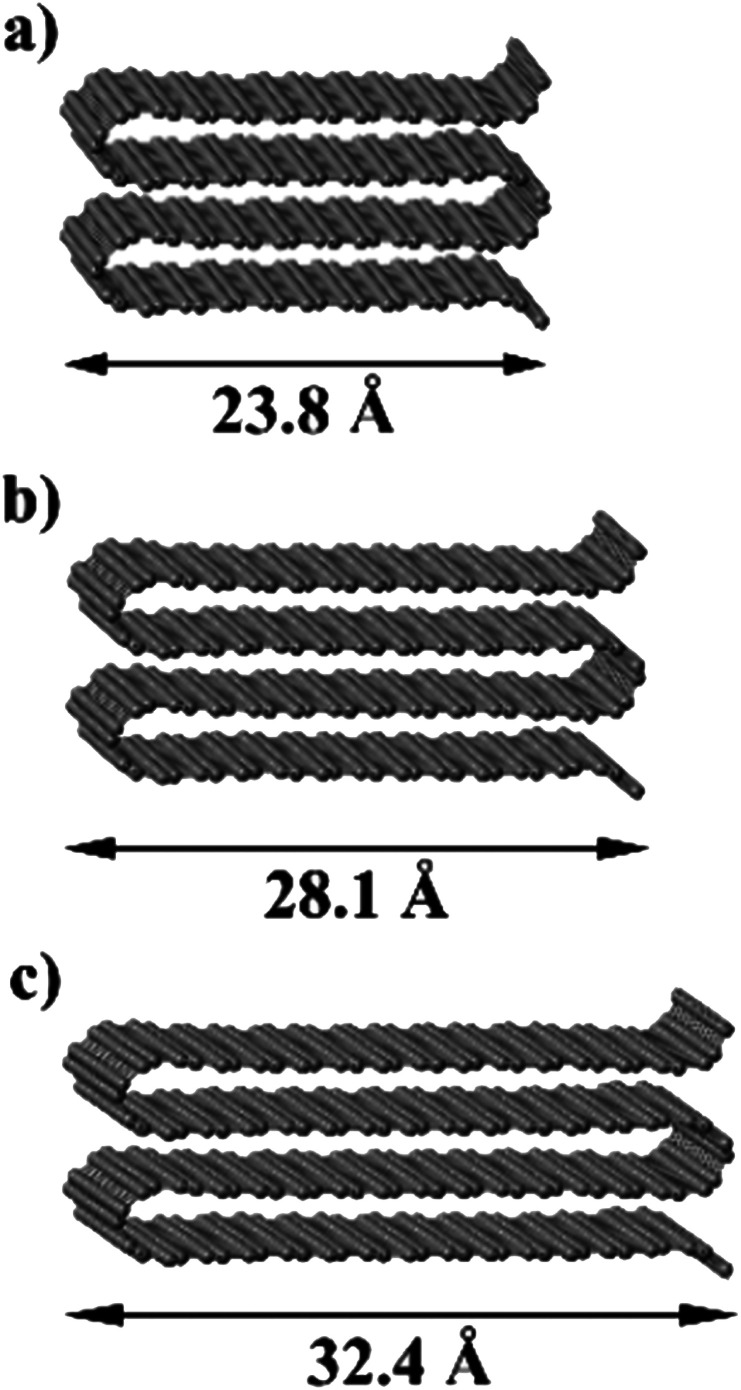
Slab models for the folded edges, including (a) 48C atoms, (b) 56C atoms, and (c) 64C atoms per simulation cell. The model is periodic in the two directions parallel to the edge plane, where a vacuum gap separates the slabs in the direction perpendicular to the edge plane.

In order to understand the thermodynamics of edge folding in graphitic structures, and in particular the formation of multiple concentric loops or folds, we resort to DFT simulations. We focus our simulations on the zigzag edge of graphite, because the geometry of the armchair edge is incompatible with the formation of a closed edge for AB stacking, as noted before by Zhan *et al.*^39^ Using the case of a single fold (*n*_f_ = 1), we first checked that our model, consisting of a continuously folded graphene sheet (Fig. 3), is long enough to represent both the closed edge and the graphite bulk, leading to converged surface energies. We consider models with 48, 56 and 64 atoms per unit cell, and found that the surface energy is indeed well converged, to *γ*_1_ = 2.99 J m^−2^, with differences between models below 0.01 J m^−2^. We are therefore confident that there is negligible interaction between the two sides of the slabs.

**Fig. 4:**
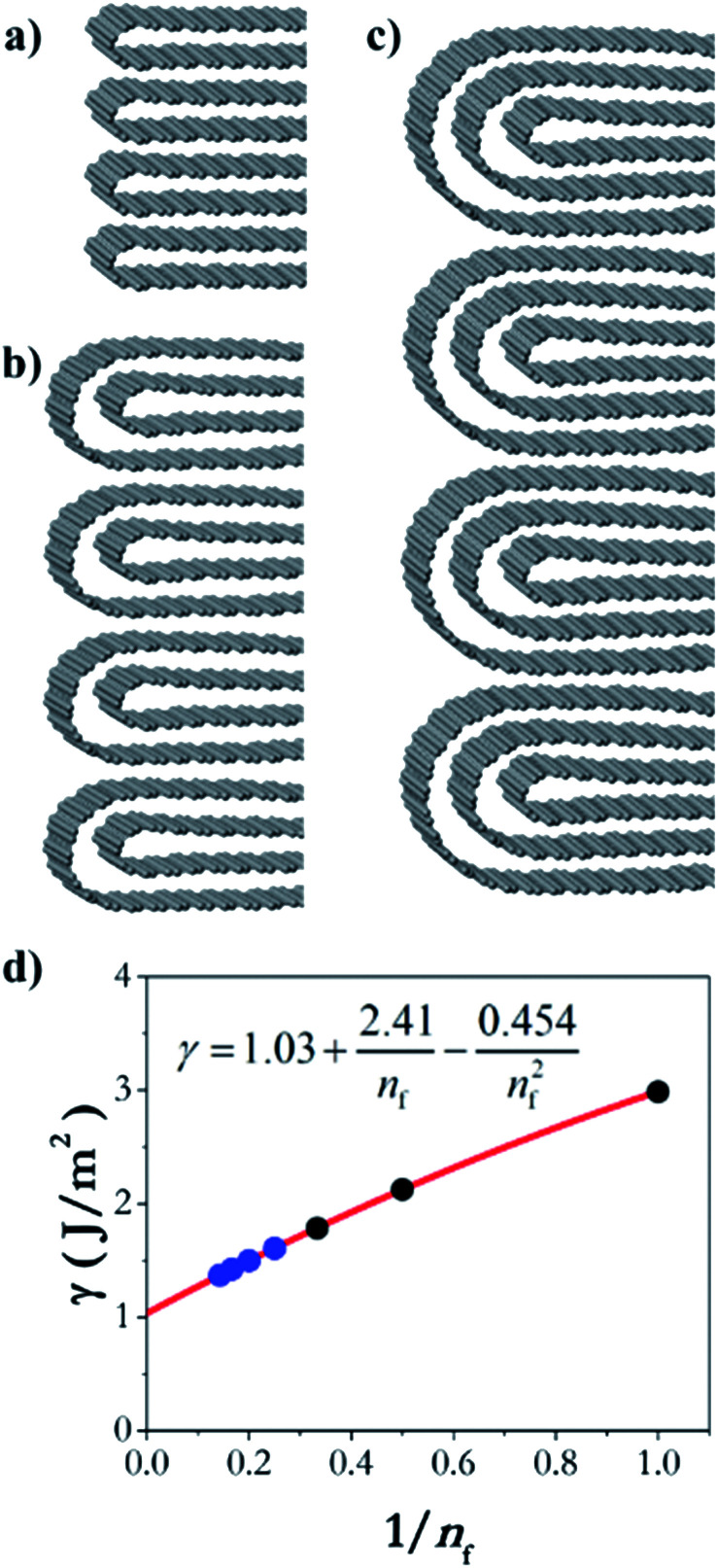
Graphitic edges with repetitions of (a) a single fold (*n*_f_ = 1), (b) two concentric folds (*n*_f_ = 2), and (c) three concentric folds (*n*_f_ = 3). (d) Variation of the surface energy *γ* with the number of concentric folds (*n*_f_). Black dots represent the values calculated directly from DFT results using eqn (1) and the blue dots and red line represent the extrapolation described in the text.

We now consider the formation of edges with two (Fig. 4b) or three (Fig. 4c) concentric folds (*n*_f_ = 2, 3). Like the single-fold edge model, these models exhibit AB stacking in the bulk region at the centre of the slabs, which remained largely unchanged upon geometry optimisation. The corresponding surface energies are *γ*_2_ = 2.13 J m^−2^ and *γ*_3_ = 1.78 J m^−2^ (for *n*_f_ = 2 and 3, respectively). It is clear that the formation of concentric loops lowers the surface energy, stabilizing the edge termination. This effect results from the smaller bending stress in the external folds, which have less curvature.

In fact, it is possible to calculate the effective strain energies *ε*_*n*_ (per unit of edge length) introduced by folds of different curvature (connecting layers which are separated by a distance (2*n* − 1)*d*, where *d* is the interlayer distance in graphite). Assuming that such strain is the dominant contribution to the surface energies, and therefore ignoring dispersion and other effects, the above calculated surface energies can be expressed in an additive way, as:2
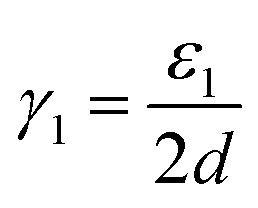
3
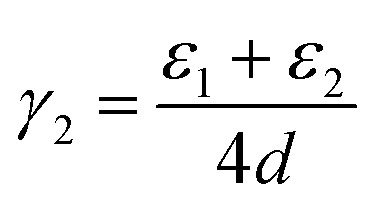
4
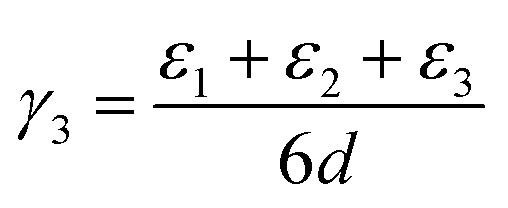


In the numerators of eqn (2)–(4), we account for the different folded edges contributing to each surface (*e.g. ε*_1_*a* is the extra energy due to the presence of a single fold), whereas in the denominator we account for the different surface areas, which are 2*da*, 4*da* and 6*da*, for *n*_f_ = 1, 2 and 3, respectively (*a* is the edge length, which cancels out from the numerator and denominator). From these expressions, we can determine the effective strain energies: *ε*_1_ = 1.25 eV Å^−1^, *ε*_2_ = 0.53 eV Å^−1^ and *ε*_3_ = 0.46 eV Å^−1^. They deviate from the pure strain values, which can be obtained from the calculated bending modulus *D* of graphene (assuming the folds are semi-cylindrical) as: π*D*/*d* = 1.48 eV Å^−1^, π*D*/3*d* = 0.49 eV Å^−1^, and π*D*/5*d* = 0.30 eV Å^−1^, respectively, because there are other contributions to the edge energy not included in the approximate model given by eqn (2)–(4) (*e.g.* dispersion interactions between the loops). Nevertheless, it is clear from the above analysis that the bending strain, which decreases with loop diameter, is the main factor driving the formation of concentric folds structures.

We can now extrapolate our results to obtain surface energies for edges consisting of arbitrary numbers of concentric folds (experimentally, it is common to find edge structures with more than three concentric folds, as shown in Fig. 2). Based on theoretical results for carbon nanotubes,^40,41^ the strain energy *ε*_*n*_ can be expected to vary linearly with the inverse of the square of the loop diameter in each fold, *d*_*n*_ = (2*n* − 1)*d*. This is indeed the case for the three values (*ε*_1_, *ε*_2_, *ε*_3_) calculated directly from DFT, and therefore the resulting surface energy *γ*_*n*_f__ can be shown to be approximately a quadratic polynomial on 1/*n*_f_ (see ESI†). Fig. 4d shows the extrapolation of the surface energies, which range between ∼3 J m^−2^ in the case of single-fold termination and ∼1 J m^−2^ in the limit of infinite concentric folds.

Whereas our analysis indicates that there is a thermodynamic preference for multiple concentric folds, in practice there will be kinetic limitations preventing the formation of such complex structures. Our analysis here is consistent with the observation by Campos-Delgado *et al.*, that when the temperature of the heat treatment of graphene nanoribbons was increased, more multiple folds formed on the edges.^42^ High temperatures are needed to provide the thermal energy to overcome the activation barriers for atomic reorganisation at the edges.

Finally, although the calculations above assume perfect graphitic structures without defects, it can be expected that the folded edges are more favourable regions for defect formation than the graphitic bulk. While it is computationally too expensive to evaluate defect formation energies in large supercells including defects at the quantum-mechanical level of calculation employed above, we have performed preliminary calculations of the segregation energies of Stones–Wales defects towards the folded edges, using the Dreiding force field^43^ as implemented in the GULP code.^44,45^ We found that Stones–Wales defects at the folded edges are much more stable than at the bulk, by up to 3.4 eV in the highest-curvature folded edge (*i.e.* the one joining two consecutive layers, and diameter *a*) and by up to 1.4 eV in the folded edge with diameter 3*a*. These energy values are only approximate due to the inaccuracies involved in the force field, but they do illustrate that there will be a considerable driving force for defects to accumulate at the folded edges of graphite, which can then be expected to be more reactive than the graphite basal planes.

### 3.3. Transformed graphite after the application of an electric field

**Fig. 5:**
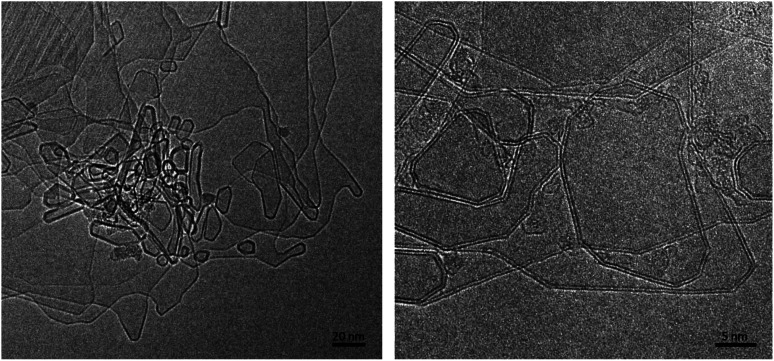
Micrographs showing transformed structures following passage of current through graphite.

We now discuss the structures formed by the application of an electric field to the closed-edge graphitic structures. The carbon collected from the graphite rods following passage of current contained some ‘normal’ graphite, but this was accompanied by many regions with a very different appearance. Typical transformed areas are shown in Fig. 5. As can be seen, the outline of the structure in this area of the material is more irregular than in the fresh graphite, with many curved and unusually-shaped features. The carbon is frequently found to be decorated with short nanotubes or nanoparticles, and in some cases, nanotube-like structures are seamlessly connected to the larger regions. These nanotube–graphene junctions have been analysed in detail in ref. 46.

**Fig. 6:**
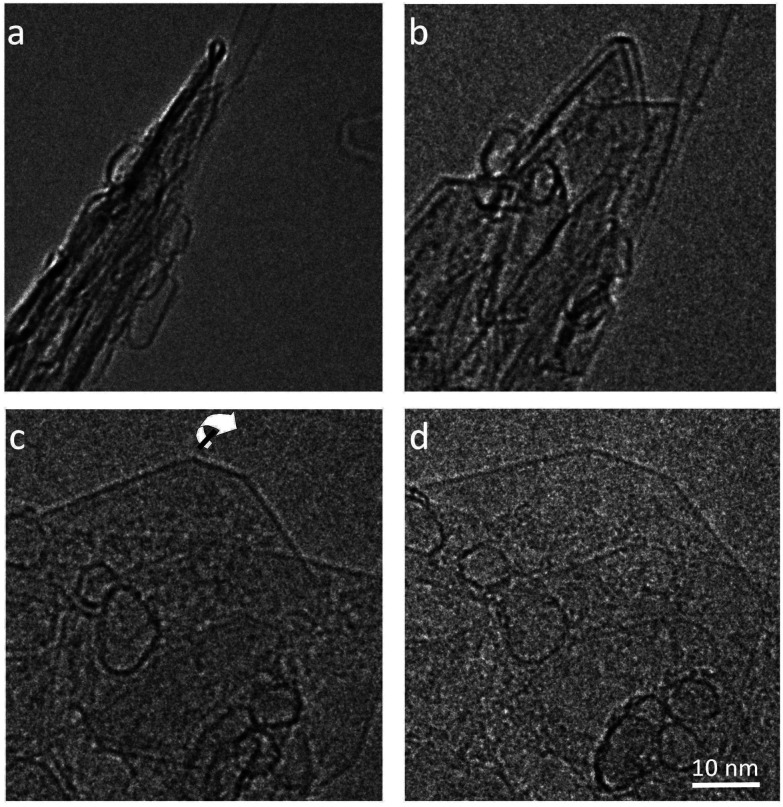
TEM images of transformed graphite at tilt values of (a) −70°, (b) −60°, (c) −30°, and (d) +40°. Arrow in (c) shows directions of tilt.

**Fig. 7:**
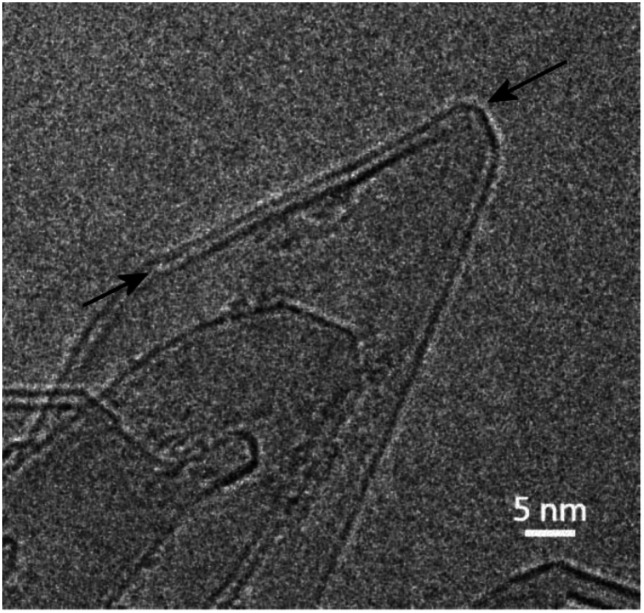
TEM image of tilted region in transformed graphite with arrows indicating expanded region along the edge.

**Fig. 8:**
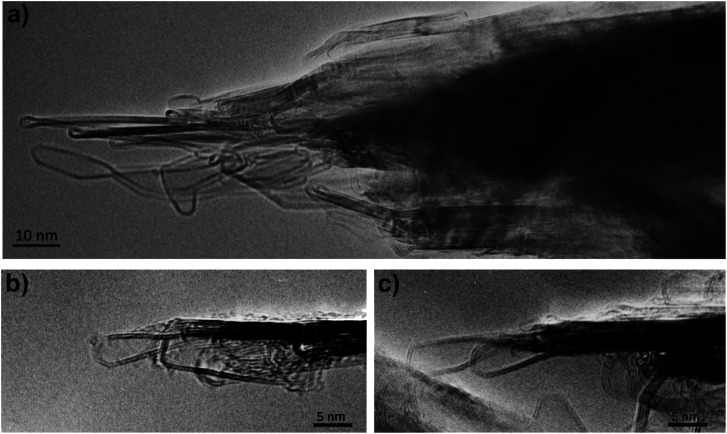
(a) Intermediate magnification image, (b) and (c) higher-magnification images of separated layers, showing bilayer structure.

In previous studies (*e.g.*ref. 13 and 14) it has been suggested that these structures are largely three-dimensional and hollow, but more recent studies using high-tilt TEM imaging have shown that many of the larger structures are flat, with a separation of the graphene layers along the edges of the crystals. Fig. 6 shows a tilt series of some typical structures. In Fig. 6a one of the structures can be seen edge-on, with a separated region clearly visible at the tip, whereas in Fig. 6b and c, the expanded regions can be seen along the margins of the particles. Another tilted region, with the expanded region along the edge indicated by arrows, is shown in Fig. 7. Some higher magnification TEM images of the separated edges are shown in Fig. 8. In the high-resolution images in Fig. 8b and c, it can be seen that the separated structures are bilayer loops attached to four layers of graphene.

It should be noted that work by Campos-Delgado *et al.*^42^ has shown that furnace annealing of graphite results in very different edge behaviour to annealing by Joule heating, *i.e.* heating by passage of a current. Furnace annealing produces edge closure, while Joule heating results in much more extensive structural transformations. The passage of a current, or the presence of an electrostatic charge, clearly has a greater effect on the structure than that produced by simple heating.

In order to understand the effect of the electric field on folded graphitic edges, we have performed DFT calculations on a single folded graphene nanoribbon, applying an electric field either parallel or perpendicular to the graphene layers. Whereas in the experiment, the graphitic layers will have random orientations with respect to the electric field, in the simulations we are considering only two limit cases for the sake of simplicity. Single-point calculations, *i.e.* without geometry relaxation, show that electronic charge redistribution takes place upon the application of the field. When the field is applied parallel to the layers, charge accumulates at the edge. However, the resulting electrostatic repulsion is not enough to separate the folded edge. In fact, geometry relaxation under the parallel field leads to minimal structural change, even when a very strong field of 3 V Å^−1^ is applied.

**Fig. 9:**
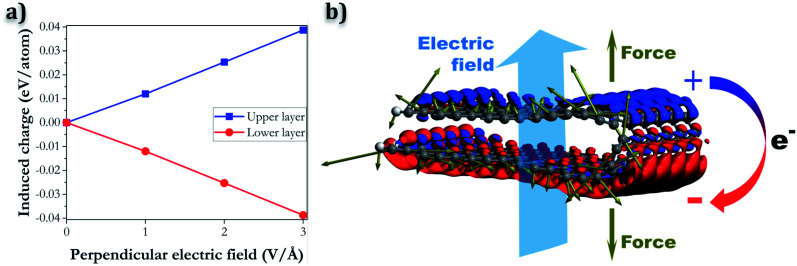
(a) Average induced charges of atoms at the upper and lower layers as function of the magnitude of the electric field applied perpendicularly and (b) charge density difference and atomic forces induced by a strong electric field in a folded nanoribbon (red and blue colours denote electron charge accumulation and depletion, respectively. The brown arrows represent the atomic forces. The example shown corresponds to an electric field of 3 V Å^−1^).

In contrast, when the electric field is applied perpendicular to the layer, a different behaviour is observed, that can explain the separation of the folded edge. In this case, the charge redistribution consists of electrons moving from one layer to the other, in the opposite direction to the electric field. For example, if we assume that the electric field is pointing upward, as in Fig. 9, negative charge will accumulate in the bottom layer, while the top layer will have a net positive charge. The charge transfer will be opposed by same-charge repulsion within a layer, and by the fact that electrons need to occupy quantum states of higher energy. The induced charges of the carbon atoms, according to a Bader charge density partition analysis,^47^ increase with the magnitude of the electric field, and are in the range of 0.01–0.04 eV per atom (see Fig. 9a). The net forces on the atoms (represented by the brown arrows in Fig. 9b) tend to pull the oppositely charged layers apart.

**Fig. 10:**
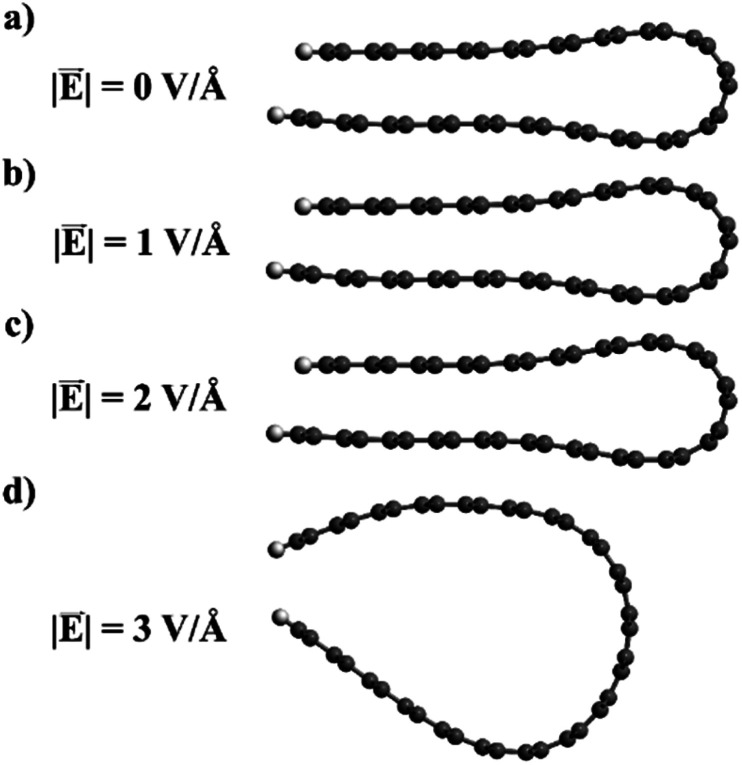
Relaxed geometries of the folded graphene ribbons in the presence of electric fields of different intensities.

Whether the folded edge expands completely or not depends on a delicate balance between the field-induced forces, the attractive dispersion forces, and the strain forces at the edge (which also tend to separate the loop). In our model nanoribbons, only the application of very strong fields (∼3 V Å^−1^) leads to loop separation upon geometry relaxation (Fig. 10). However, the field strength required to separate the folded edges in real graphitic systems can be expected to be very different, since other factors, like temperature or the presence of defects, might play a role not accounted for in our nanoribbon model. For example, in the experiments described above in this paper, the graphitic system is heated above 2000 K, so kinetic barriers preventing the expansion of the loop edge can be easily overcome. In contrast, our model is static and essentially represents the system at 0 K; therefore it is not surprising that an unphysically large electric field is required to reproduce an effect observed at much larger temperatures. Our calculations provide only qualitative insight into the behaviour of the closed edges in the presence of an electric field, but the proposed picture is physically reasonable and in good agreement with the experimental observation.

## 4. Conclusions

We have presented an experimental and theoretical investigation of the folded edge structure of graphite, including an analysis of the thermodynamics of edge folding and the effect of an electric field. Our density functional theory calculations explain the preference for multiple concentric loops at the edges observed by transmission electron microscopy, and quantify the stability of different edge terminations as a function of the number of concentric loops.

We also report transmission electron microscopy images of the transformed structures obtained by the application of an electric field, and show that this could lead to the separation of the closed edges. We propose a model to explain this behaviour based on density functional theory calculations. The electric field leads to a charge redistribution resulting in layers with opposite charge, which are pulled apart by the electric field. Geometry relaxation of folded graphene nanoribbons in the presence of an electric field confirms the tendency to loop expansion.

Our study contributes both experimental evidence and new theoretical insights into the properties of graphite closed edges, and their response to electric fields. The results will help to explain the effect of electric fields on the structure of graphitic carbons, and may have implications for understanding the growth of carbon nanotubes by arc-discharge.

## Conflicts of interest

There are no conflicts of interest to declare.

## Supplementary Material

RA-010-C9RA09913A-s001

## References

[cit1] Harris P. J. F. (2018). Transmission Electron Microscopy of Carbon: A Brief History. C.

[cit2] Buseck P. R., Bo-Jun H., Keller L. P. (1987). Electron Microscope Investigation of the Structures of Annealed Carbons. Energy Fuels.

[cit3] Rotkin S., Gogotsi Y. (2002). Analysis of non-planar graphitic structures: from arched edge planes of graphite crystals to nanotubes. Mater. Res. Innovations.

[cit4] Liu Z., Suenega K., Harris P. J. F., Iijima S. (2009). Open and Closed Edges of Graphene Layers. Phys. Rev. Lett..

[cit5] Acik M., Chabal Y. J. (2011). Nature of Graphene Edges: A Review. Jpn. J. Appl. Phys..

[cit6] Zhang X., Xin J., Ding F. (2013). The edges of graphene. Nanoscale.

[cit7] Yan J. (2016). *et al.*, Graphene homojunction: closed-edge bilayer graphene by pseudospin interaction. Nanoscale.

[cit8] Huang J. Y. (2009). *et al.*, In situ observation of graphene sublimation and multi-layer edge reconstructions. Proc. Natl. Acad. Sci. U. S. A..

[cit9] Chopra N. G. (1995). *et al.*, Fully collapsed carbon nanotubes. Nature.

[cit10] Machon D. (2018). *et al.*, Perspective: high pressure transformations in nanomaterials and opportunities in material design. J. Appl. Phys..

[cit11] Balima F. (2016). *et al.*, Radial collapse of carbon nanotubes for conductivity optimized polymer composites. Carbon.

[cit12] Harris P. J. F. (2009). Ultrathin graphitic structures and carbon nanotubes in a purified synthetic graphite. J. Phys.: Condens. Matter.

[cit13] Harris P. J. F. (2012). Hollow structures with bilayer graphene walls. Carbon.

[cit14] Harris P. J. F. (2014). *et al.*, Bilayer graphene formed by passage of current through graphite: evidence for a three-dimensional structure. Nanotechnology.

[cit15] Harris P. J. F. (2016). Structural transformation of natural graphite by passage of an electric current. Carbon.

[cit16] Jia X. (2009). *et al.*, Controlled Formation of Sharp Zigzag and Armchair Edges in Graphitic Nanoribbons. Science.

[cit17] Qi L. (2010). *et al.*, In situ observations of the nucleation and growth of atomically sharp graphene bilayer edges. Carbon.

[cit18] Girifalco L. A., Hodak M. (2002). van der Waals binding energies in graphitic structures. Phys. Rev. B: Condens. Matter Mater. Phys..

[cit19] Barreiro A. (2012). *et al.*, Graphene at High Bias: Cracking, Layer by Layer Sublimation, and Fusing. Nano Lett..

[cit20] Harris P. J. F. (2017). Engineering carbon materials with electricity. Carbon.

[cit21] Alyobi M. M. M. (2019). *et al.*, Modifying the electrical properties of graphene by reversible point-ripple formation. Carbon.

[cit22] Lopez-Bezanilla A. (2012). *et al.*, Geometric and Electronic Structure of Closed Graphene Edges. J. Phys. Chem. Lett..

[cit23] Ugarte D. (1992). Curling and closure of graphitic networks under electron-beam irradiation. Nature.

[cit24] Kresse G., Furthmüller J. (1996). Efficiency of ab-initio total energy calculations for metals and semiconductors using a plane-wave basis set. Comput. Mater. Sci..

[cit25] Kresse G., Furthmüller J. (1996). Efficient iterative schemes for ab initio total-energy calculations using a plane-wave basis set. Phys. Rev. B: Condens. Matter Mater. Phys..

[cit26] Berland K. (2015). *et al.*, van der Waals forces in density functional theory: a review of the vdW-DF method. Rep. Prog. Phys..

[cit27] Klimeš J., Bowler D. R., Michaelides A. (2010). Chemical accuracy for the van der Waals density functional. J. Phys.: Condens. Matter.

[cit28] Klimeš J., Bowler D. R., Michaelides A. (2011). van der Waals density functionals applied to solids. Phys. Rev. B: Condens. Matter Mater. Phys..

[cit29] Ontaneda J. (2018). *et al.*, Origin of the monolayer Raman signature in hexagonal boron nitride: a first-principles analysis. J. Phys.: Condens. Matter.

[cit30] Pykal M. (2016). *et al.*, Modelling of graphene functionalization. Phys. Chem. Chem. Phys..

[cit31] Wu X. (2001). *et al.*, Towards extending the applicability of density functional theory to weakly bound systems. J. Chem. Phys..

[cit32] Klimeš J., Michaelides A. (2012). Perspective: advances and challenges in treating van der Waals dispersion forces in density functional theory. J. Chem. Phys..

[cit33] Grimme S. (2006). Semiempirical GGA-type density functional constructed with a long-range dispersion correction. J. Comput. Chem..

[cit34] Grimme S. (2010). *et al.*, A consistent and accurate ab initio parametrization of density functional dispersion correction (DFT-D) for the 94 elements H-Pu. J. Chem. Phys..

[cit35] Torres-Dias A. C. (2017). *et al.*, From mesoscale to nanoscale mechanics in single-wall carbon nanotubes. Carbon.

[cit36] Blöchl P. E. (1994). Projector augmented-wave method. Phys. Rev. B: Condens. Matter Mater. Phys..

[cit37] Kresse G., Joubert D. (1999). From ultrasoft pseudopotentials to the projector augmented-wave method. Phys. Rev. B: Condens. Matter Mater. Phys..

[cit38] Santos E. J. G., Kaxiras E. (2013). Electric-Field Dependence of the Effective Dielectric Constant in Graphene. Nano Lett..

[cit39] Zhan D., Liu L., Xu Y. N., Ni Z. H., Yan J. X., Zhao C., Shen Z. X. (2011). Low temperature edge dynamics of AB-stacked bilayer graphene: naturally favored closed zigzag edges. Sci. Rep..

[cit40] Xin Z., Jianjun Z., Zhong-can O.-Y. (2000). Strain energy and Young's modulus of single-wall carbon nanotubes calculated from electronic energy-band theory. Phys. Rev. B: Condens. Matter Mater. Phys..

[cit41] Robertson D. H., Brenner D. W., Mintmire J. W. (1992). Energetics of nanoscale graphitic tubules. Phys. Rev. B: Condens. Matter Mater. Phys..

[cit42] Campos-Delgado J. (2009). *et al.*, Thermal stability studies of CVD-grown graphene nanoribbons: defect annealing and loop formation. Chem. Phys. Lett..

[cit43] Mayo S. L., Olafson B. D., Goddard III W. A. (1990). DREIDING: A Generic Force Field for Molecular Simulations. J. Phys. Chem..

[cit44] Gale J. D. (1997). GULP: a computer program for the symmetry-adapted simulation of solids. J. Chem. Soc., Faraday Trans..

[cit45] Gale J. D., Rohl A. L. (2003). The General Utility Lattice Program (GULP). Mol. Simul..

[cit46] Harris P. J. F., Suarez-Martinez I., Marks N. A. (2016). The structure of junctions between carbon nanotubes and graphene shells. Nanoscale.

[cit47] Bader R. F. W. , Atoms in Molecules: A Quantum Theory, Oxford University Press, 1994

