# Supplementary material for: The closed-edge structure of graphite and the effect of electrostatic charging
Source: RSC Adv. 2020 Feb 24;10(13):7994–8001. doi: 10.1039/c9ra09913a (PMC9049950; doi:10.1039/c9ra09913a)
Supplement: RA-010-C9RA09913A-s001 [file RA-010-C9RA09913A-s001.pdf]

Electronic Supplementary Material (ESI) for RSC Advances.
This journal is © The Royal Society of Chemistry 2020

The closed-edge structure of graphite and the effect of electrostatic charging

Supplementary Information

Victor Posligua[a], Joana Bustamante[b], Cesar H. Zambrano[c], Peter J. F. Harris[d], and Ricardo Grau-Crespo[a*]

[a]Department of Chemistry, University of Reading, Whiteknights, Reading RG6 6AD, United Kingdom.
[b]Departamento de Química, Universidad Técnica Particular de Loja, San Cayetano Alto, Loja 1101608, Ecuador.
[c]Instituto de Simulación Computacional (ISC-USFQ), Diego de Robles y Vía Interoceánica, Universidad San Francisco de Quito, 17-1200-841 Quito, Ecuador.
[c]Electron Microscopy Laboratory, J. J. Thomson Building, University of Reading, Whiteknights, Reading RG6 6AF, United Kingdom.

1. Bending modulus of graphene from DFT/optPBE-vdW

We have calculated the bending modulus *D* of graphene from the DFT energies (using the optPBE-vdW functional) of three zigzag nanotubes of different sizes, with chiral indices (14,0), (16,0) and (18,0). The variation of the energy (per C atom) of the nanotubes with the radii is approximately given by the equation:

$$E(R) = E(\infty) \quad + \frac{D\,s}{2\,R^2}$$

where $E(\infty)$ is the energy (per C atom) in the zero-curvature limit, and *s* is the surface area per C atom in graphene. The bending modulus can be extracted from the plot shown in **Figure S1**, and we obtain *D*=1.57 eV, in good agreement with the experimental value of 1.7±0.2 eV [1].

* Corresponding Author: r.grau-crespo@reading.ac.uk

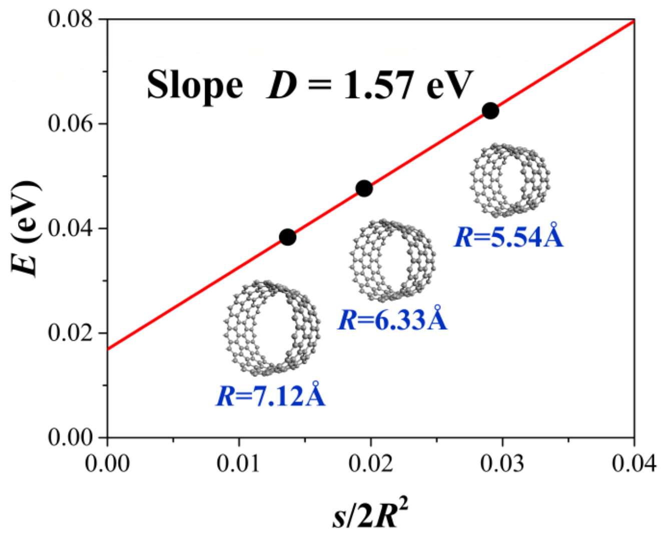

Figure S1. Calculation of the bending modulus of graphene from the DFT energies of nanotubes of different sizes.

2. Extrapolation of surface energies of closed edges

In order to extrapolate the surface energies (γ) to an arbitrary number (n_f) of concentric folds (**Figure 4d** in the article), we assume that the strain energy ε_n varies linearly with the inverse of the square of the loop diameter, which corresponds to "opening distance" (d_n = $(2n$-$1)d$) of the fold. This is valid for the three points obtained from the DFT simulations, as shown in **Figure S**, and is consistent with the observed variation of strain energy in carbon nanotubes as a function of size.

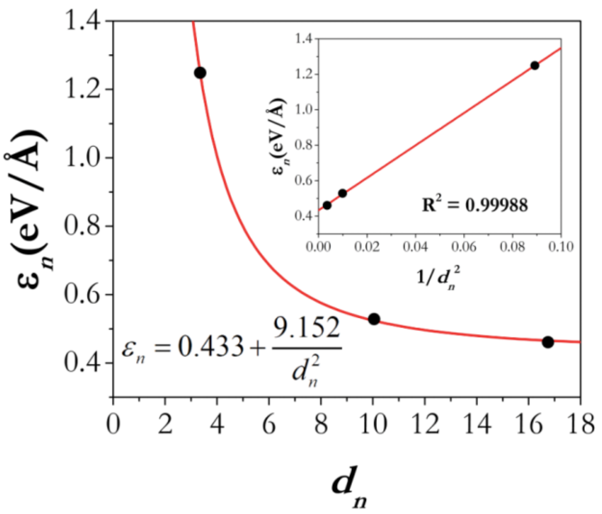

Figure S2. Variation of the strain energy with the loop diameter ("opening distance" d_n).

We now write this dependence as:

$$\varepsilon_n = \alpha + \frac{\beta}{d_n^2}$$

where α = 0.433 and β = 9.152 from the fitting of the DFT data (**Figure S2**). The surface energy of the edge consisting of a periodic repetition of n_f concentric folds can then be calculated as:

$$\gamma_{n_\mathrm{f}} = \frac{1}{2n_\mathrm{f}d}\sum_{n=1}^{n_\mathrm{f}}\left(\alpha + \frac{\beta}{(2n-1)^2 d^2}\right)$$

$$= \frac{1}{2n_\mathrm{f}d}\left(\alpha n_\mathrm{f} + \frac{\beta}{d^2}\sum_{n=1}^{n_\mathrm{f}}\frac{1}{(2n-1)^2}\right)$$

$$= \frac{\alpha}{2d} + \frac{\beta}{2d^3 n_\mathrm{f}} f(n_\mathrm{f})$$

where:

$$f(n_\mathrm{f}) = \sum_{n=1}^{n_\mathrm{f}}\frac{1}{(2n-1)^2} \approx \frac{\pi^2}{8} - \frac{\zeta}{n_\mathrm{f}}$$

and $\zeta \approx 0.2384$. The validity of the approximate expression above is clear from **Figure S3**.

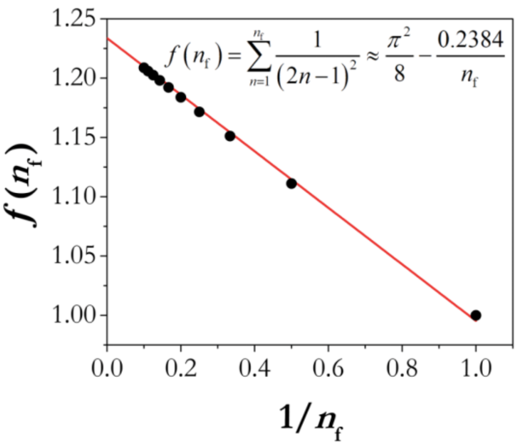

Figure S3. Quasi-linear variation of function $f(n_f)$ with $1/n_f$.

Using these approximations, the expression for the surface energy is:

$$\gamma_{n_{\mathrm{f}}} = \frac{\alpha}{2d} + \frac{\pi^2 \beta}{16 d^3 n_{\mathrm{f}}} - \frac{\beta \zeta}{2 d^3 n_{\mathrm{f}}^2}$$

which represents a second-order polynomial in $1/n_f$. We use this equation to extrapolate our DFT-calculated surface energies to edges with higher numbers of concentric folds (**Figure 4d** in the article).

3. Additional TEM images

Figure S4 shows additional TEM images of expanded edges in graphite exposed to electric field.

Figure S5 illustrates the effect of intense electron irradiation, with an electron flux 10 - 20 times higher than what would normally be used for imaging. This was achieved by removing the condenser aperture and focussing the beam on to the crystal. It can be seen that the structure has been completely disrupted, with some evidence of curling of the graphitic planes. Such disruption was never seen during normal imaging.

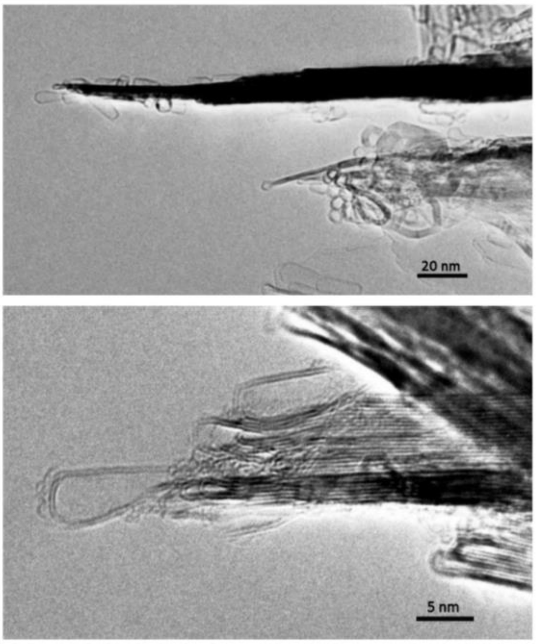

Figure S4. Additional TEM images of expanded edges in graphite exposed to electric field.

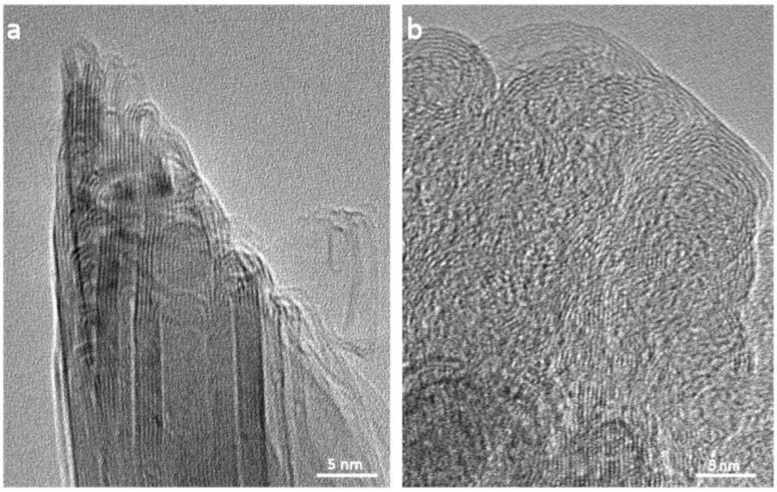

Figure S5. TEM images illustrating the damaging effect of intense electron irradiation. (a) Original structure; (b) Structure observed after exposure to the intense beam.

References

1. Torres-Dias, A.C., et al., *From mesoscale to nanoscale mechanics in single-wall carbon nanotubes.* Carbon, 2017. **123**: p. 145-150.
